# Unlocking potential: low frequency subthalamic nucleus stimulation enhances executive function in Parkinson’s disease patients with postural instability/gait disturbance

**DOI:** 10.3389/fnins.2023.1228711

**Published:** 2023-08-30

**Authors:** Guofan Qin, Hutao Xie, Lin Shi, Baotian Zhao, Yifei Gan, Zixiao Yin, Yichen Xu, Xin Zhang, Yaojing Chen, Yin Jiang, Quan Zhang, Jianguo Zhang

**Affiliations:** ^1^Department of Neurosurgery, Beijing Tiantan Hospital, Capital Medical University, Beijing, China; ^2^Beijing Key Laboratory of Neurostimulation, Beijing, China; ^3^Department of Functional Neurosurgery, Beijing Neurosurgical Institute, Capital Medical University, Beijing, China; ^4^State Key Laboratory of Cognitive Neuroscience and Learning, Beijing Normal University, Beijing, China

**Keywords:** Parkinson’s disease, postural instability/gait disturbance, subthalamic nucleus, deep brain stimulation, low frequency, cognition, executive function, conflict resolution

## Abstract

Postural instability/gait disturbance (PIGD) is very common in advanced Parkinson’s disease, and associated with cognitive dysfunction. Research suggests that low frequency (5–12 Hz) subthalamic nucleus-deep brain stimulation (STN-DBS) could improve cognition in patients with Parkinson’s disease (PD). However, the clinical effectiveness of low frequency stimulation in PIGD patients has not been explored. This study was designed in a double-blinded randomized cross-over manner, aimed to verify the effect of low frequency STN-DBS on cognition of PIGD patients. Twenty-nine PIGD patients with STN-DBS were tested for cognitive at off (no stimulation), low frequency (5 Hz), and high frequency (130 Hz) stimulation. Neuropsychological tests included the Stroop Color-Word Test (SCWT), Verbal fluency test, Symbol Digital Switch Test, Digital Span Test, and Benton Judgment of Line Orientation test. For conflict resolution of executive function, low frequency stimulation significantly decreased the completion time of SCWT-C (*p* = 0.001) and Stroop interference effect (*p* < 0.001) compared to high frequency stimulation. However, no significant differences among stimulation states were found for other cognitive tests. Here we show, low frequency STN-DBS improved conflict resolution of executive function compared to high frequency. Our results demonstrated the possibility of expanding the treatment coverage of DBS to cognitive function in PIGD, which will facilitate integration of low frequency stimulation into future DBS programming.

## 1. Introduction

Parkinson’s disease (PD) is a multifaceted neurodegenerative disorder characterized by a wide range of motor and non-motor symptoms. Subtypes of PD have been identified, with patients categorized based on unique clinical characteristics, such as postural instability/gait disturbance (PIGD) subtypes and tremor-dominant (TD) (Thenganatt and Jankovic, 2014; Tsuboi et al., 2020).

PIGD represents the predominant motor subtype in PD, accounting for more than half of the deep brain stimulation (DBS) candidates (Thenganatt and Jankovic, 2014; Katz et al., 2015; Nutt, 2016; Fan et al., 2022). Patients with PIGD are associated with not only more rapid progression of motor dysfunction, but also higher risks of cognitive impairment (especially executive function), and more possibility to develop dementia (Alves et al., 2006; Burn et al., 2006; Amboni et al., 2013; Kelly et al., 2015; Arie et al., 2017). Since the similar neural substrates have been found to be involved in both increased motor severity and cognitive decline, which may lead to a higher risk of developing dementia at an earlier age (Aarsland et al., 2003; Alves et al., 2006; Burn et al., 2006; Janvin et al., 2006; Williams-Gray et al., 2007). Cholinergic deficiencies in the pedunculopontine nucleus and neocortex may contribute to postural instability and gait disturbances in Parkinson’s disease (Bohnen et al., 2009), while deficits in basal forebrain nuclei, prefrontal and temporal regions may impair attentional/executive function and memory (Yarnall et al., 2011). The cognitive dysfunction could result in recurrent falls, increased risk of hospitalization, disability and even death, which will significantly compromise the patient’s quality of life and cause a heavy burden on the caregivers (Fasano et al., 2010; Simuni et al., 2016; Fasano et al., 2017). Therefore, interventions aimed at addressing cognitive impairment in patients with PIGD are equally crucial as those targeting motor symptoms.

DBS is a safe and effective neurosurgical intervention that provides an alternative therapeutic option for patients with advanced disease who experience inadequate symptom control and compromised quality of life despite optimal medication management (Deuschl et al., 2006; Hariz and Blomstedt, 2022). High frequency (100–180 Hz) DBS in the subthalamic nucleus (STN) has been proved to be an effective treatment for motor symptoms in patients with PIGD (Katz et al., 2015; Shin et al., 2020; Fan et al., 2022). The oscillatory mechanism indicates that high-frequency STN-DBS promotes beta power desynchronization and gamma power synchronization in the basal ganglia-thalamocortical motor network, which is associated with the observed clinical benefits (Eusebio et al., 2012; Cao et al., 2017; Müller and Robinson, 2018). Nevertheless, extensive data has revealed the declines in cognitive function in PD patients after long-term high frequency STN-DBS, involving executive function, memory, and language (Parsons et al., 2006; Temel et al., 2006; Fasano et al., 2010; Xie et al., 2022), which may further increase unfavorable cognitive effects of PIGD patients. There is growing evidence to suggest that the low-frequency oscillations (LFOs,5–12 Hz) in the subthalamic nucleus and its connections to the cortex, especially the medial prefrontal cortex (mPFC) (Kelley et al., 2018; Zavala et al., 2018), are integral to cognitive function (Wojtecki et al., 2006; Witt et al., 2008; Brittain et al., 2012). Accordingly, an accumulating body of evidence suggests that low frequency STN-DBS (5–12 Hz) may improve cognitive function of PD patients (Wojtecki et al., 2006; Scangos et al., 2018; Lam et al., 2021; Lee et al., 2021). More specifically, low frequency stimulation improves verbal fluency compared to high frequency stimulation (Wojtecki et al., 2006; Lam et al., 2021; Lee et al., 2021), and enhances cognitive control compared to no stimulation (Scangos et al., 2018).

However, considering that the correlation of PIGD with more severe cognitive deficits, there is no research to explore the effect of low-frequency stimulation on cognitive function of patients with PIGD. In addition, some confounders in previous studies impede the clinical reliability of low frequency stimulation, such as the potential impact of long-term high frequency stimulation prior to the trial (≥ 3 months) (Wojtecki et al., 2006; Lam et al., 2021), relatively short stimulation and washout periods (5–30 min) (Wojtecki et al., 2006; Lam et al., 2021; Lee et al., 2021), and interference of anti-PD medication during cognition evaluation (Scangos et al., 2018; Lam et al., 2021; Lee et al., 2021).

In the current study, we aimed to reveal the effects of low frequency stimulation on cognition of PIGD patients with confounders well controlled. We conducted a prospective double-blinded randomized clinical trial to compare the effects of bilateral STN low and high frequency stimulation on all cognitive domains during their initial programming, with prolonged stimulation and washout time and exclusion of anti-PD medication interference.

## 2. Materials and methods

This study was designed in a double-blinded, randomized, cross-over manner. In accordance with the experimental design employed in previous studies, each patient within a single group was exposed to distinct stimulation states arranged in a unique sequence (Scangos et al., 2018; Lam et al., 2021; Lee et al., 2021). The present study was approved by the local Institutional Review Board (IRB) of Beijing Tiantan Hospital (IRB number: KY 2018-008-01), and registrated at China Clinical Trials Center (www.chictr.org.cn, ChiCTR2100044479; March 22, 2021). Informed consent was provided by all patients according to the Declaration of Helsinki.

### 2.1. Participants and surgery

Thirty-two patients diagnosed with idiopathic PD underwent comprehensive preoperative clinical assessment, including neurology, neurosurgery, and neuropsychology. These patients were prospectively enrolled and subsequently received STN-DBS implantation after obtaining informed consent. Inclusion criteria were: (1) age between 45 and 75 years; (2) patients underwent bilateral STN-DBS; (3) belonged to the PIGD subtype. The ratio of mean tremor scores and mean postural instability/gait disturbance scores in MDSUPDRS was used to define PIGD (ratio ≤ 0.9) (Stebbins et al., 2013). Exclusion criteria included dementia, severe psychiatric disturbances, vision and hearing abnormalities, history of stroke, and brain tumor/traumatic surgery. The clinical assessment involved the collection of variables including sex, age, disease duration, years of education and levodopa equivalent dose. The Mini-Mental State Examination (MMSE) was employed for the comprehensive evaluation of patients’ cognitive function. The Hamilton Anxiety Scale (HAMA, 14-item version) was utilized to assess the anxiety status of patients. Additionally, the Hamilton Depression Scale (HAMD, 24-item version) was employed to evaluate depressive status in patients. MDSUPDRS III score was preoperatively evaluated in both the medication withdrawal (medoff) stage and the medication (med-on) stage. The medication response was calculated as follows:


$$\begin{array}{l} Medication\\ response=\frac{{\left(\begin{array}{l} MDS-\\ UPDRS\;III \end{array}\right)}_{med-off}-{\left(\begin{array}{l} MDS-\\ UPDRS\;III \end{array}\right)}_{med-on}}{{\left(\begin{array}{l} MDS-\\ UPDRS\;III \end{array}\right)}_{med-off}}\times 100\% \end{array}$$

**Figure 1:**
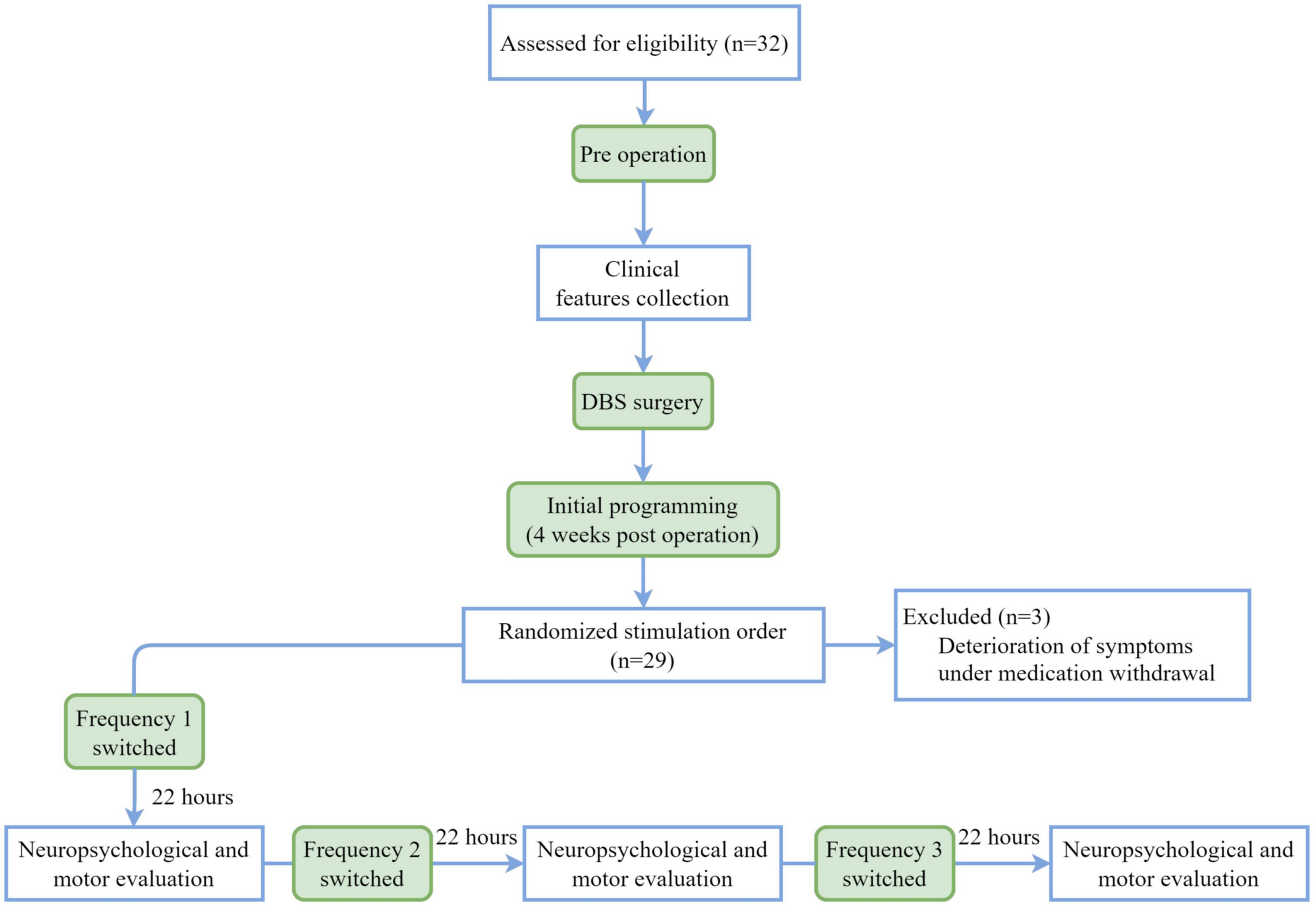
Study flowchart.

All operations were performed by the same neurosurgical team. Briefly, patients underwent bilateral STN-DBS under local anesthesia. Surgeries were performed using a Leksell G frame system with the assistance of a Leksel Surgiplan workstation (Elekta Instrument AB, Stockholm, Sweden). Microelectrode recordings and macrostimulation were used to accurately target the STN. Quadripolar DBS electrodes (Model L301; PINS Medical Co. Ltd.) were implanted and fixed, and the internal pulse generator was implanted subsequently. After 4 weeks, patients returned to the hospital and the experiment was started in order to minimize brain microlesion effects and emotional fluctuations caused by surgery. The study flowchart is outlined in Figure 1.

### 2.2. DBS electrode localization

**Figure 2:**
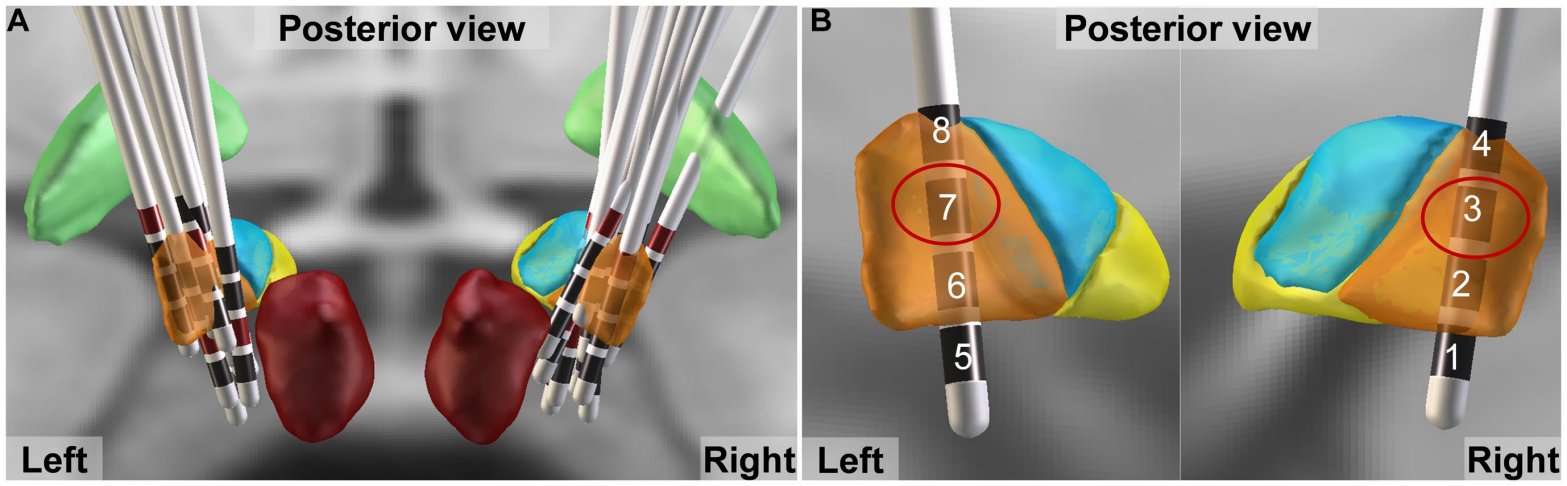
DBS electrode localization. **(A)** Lead trajectories and contacts of all patients. Globus pallidus internus, and red nucleus are shown as green and dark red, respectively. The motor, associative and limbic areas of subthalamic nucleus (STN) are shown as orange, blue and yellow, respectively. **(B)** The activated contacts closest to the dorsolateral STN (motor area) are shown by red circle.

The DBS electrode positions were confirmed with the LeadDBS toolbox (Version 2.5.3; www.leaddbs.org) based on preoperative MRI and postoperative CT, as previously described (Horn et al., 2019). Briefly, postoperative CT scans were linearly coregistered to the preoperative MRI (MP-RAGE sequence) using the Advanced Normalization Tools (ANTs; http://stnava.github.io/ANTs/). Registration between postoperative CT and preoperative MRI was further refined with the “brain shift correction” module, which focuses on the subcortical target region of interest and thus minimizes nonlinear bias introduced when opening the skull during surgery. The images were normalized to standard Montreal Neurological Institute space with the symmetric diffeomorphic registration algorithm implemented in the ANTs. Lead trajectories and contacts were automatically prelocalized with PaCER and manually refined. The DISTAL Minimal atlas was used to synthesize 3D views of electrodes and nucleus (Figure 2A) (Ewert et al., 2018).

### 2.3. Stimulation parameters

We evaluated three stimulation states during patients’ initial programming. Given that the dorsolateral STN serves as the sensorimotor region of STN and is commonly targeted for motor symptom improvement in PD through DBS (Bot et al., 2018; Dembek et al., 2019), the active contact closest to this area was selected based on electrode localization results obtained from the Lead-DBS toolbox (Figure 2B), prioritizing enhancement of motor symptoms. The stimulation order of off (no stimulation), low frequency, and high frequency was randomized to counterbalance learning effects of cognitive tests, which was blinded to the evaluators and patients. A low frequency of 5 Hz was employed, based on previous studies utilizing settings within the range of 5-12 Hz to enhance cognitive function in Parkinson’s disease (Wojtecki et al., 2006; Scangos et al., 2018; Lam et al., 2021; Lee et al., 2021), while a high frequency of 130 Hz was utilized as it is the most commonly used parameter for improving motor symptoms in clinical practice (Moro et al., 2002; Dayal et al., 2017). Then the current intensity and pulse width mostly suitable for relieving motor symptoms were determined when the stimulation frequency was adjusted to 130 Hz, so as to give priority to the improvement of movement. Then one neurologist unblinded to the stimulation order conducted frequency adjustment with monopolar electrical configuration, constant current intensity (mA) and pulse width (μs). Each stimulation state lasted for 22 h. After neuropsychological and motor evaluation the frequency was switched to ensure sufficient stimulation and washout period (Figure 1).

### 2.4. Neuropsychological and motor evaluation

In accordance with prior investigations on cognitive function domains in PD patients, our chosen neuropsychological tests comprehensively encompassed each domain, including executive function, language, attention, short-term memory, and visuospatial ability (Abboud et al., 2015; Aarsland et al., 2021). Conflict resolution of executive function was evaluated by the Stroop Color-Word Test (SCWT) (Stroop, 1935; Bryan and Luszcz, 2000). It was administered in Chinese and each card contained 50 items. With card A, the color names had to be read as quickly and as accurately as possible; with card B, the color of dots had to be named as quickly and as accurately as possible; and with card C, the ink colors of colored names that were incongruent had to be named (Chen et al., 2019). The task accuracy for cards A, B, and C were recorded as SCWT-A_accuracy_, SCWT-B_accuracy_, and SCWT-C_accuracy_. The completion time for cards A, B, and C were recorded as SCWT-A_time_, SCWT-B_time_, and SCWT-C_time_. Stroop interference effect (SIE) was calculated as follows: SIE = SCWT-C_time_ − [(SCWT-A_time_ + SCWT-B_time_)/2] (van der Elst et al., 2006). Language was evaluated by the Verbal fluency test (VFT) (Fernández et al., 2002; Zhao et al., 2013), consisting of an animal fluency test (AFT, representing the episodic category), a household items fluency test (HFT, representing the non-episodic category), and switching fluency test between above two categories. The patients were instructed to generate as many words as possible within a 60-s timeframe that corresponded to the given category, and their score was determined by the number of appropriate words produced. Attention was evaluated by the oral Symbol Digital Switch Test (SDMT) (Ma et al., 2019). The patients were required to match symbols with their corresponding numbers within a 90-s time frame, and the score were determined by the number of correct matches made. Short-term memory was evaluated by the Digital Span Test (DST), consisting of forward and backward digits (Woods et al., 2011). The patients were required to recite, in order or reverse order, a string of numbers from the evaluator, with the correct entry as the score. Visuospatial ability was evaluated by the Benton Judgment of Line Orientation (JLO) test (Benton et al., 1978). The patients were instructed to identify the number of lines present on a semicircle at various angles, and the accurate responses were recorded as a score.

We also assessed motor function using MDS-UPDRS III. Patients underwent neuropsychological and motor evaluations postoperatively for three stimulation states on three consecutive days. Cognitive tests were evaluated by one physician who had received neuropsychological training. Another physician evaluated motor function. Both were masked to the stimulation order of patients. Testing started at 9 a.m. each day and lasted almost 1 h. To control for potential confounding effects of medication on cognition, the cognitive assessments of all patients were performed at least after 12 h withdrawal of anti-Parkinson medication.

### 2.5. Statistical analysis

Statistical analysis was performed with GraphPad Prism 9.2.0 (GraphPad Software, San Diego, California United States). The Shapiro–Wilk test was used to test for normality. We compared no stimulation, low frequency stimulation, and high frequency stimulation using a repeated-measures analysis of variance (rmANOVA) for each cognitive test and motor scores, followed by Tukey multiple comparisons tests. Mauchly’s test was used to test for sphericity; Greenhouse–Geisser correction was used if sphericity was not assumed. Differences were considered statistically significant if two-tailed *p* < 0.05.

## 3. Results

Three of 32 patients withdrew from the trial because they could not tolerate deterioration of symptoms under anti-PD medication withdrawal. As a result, 29 PIGD patients (17 males, mean age:62.7 years, range 48–79 years) were included in the final analysis. The demographics and clinical features are detailed in Table 1 and DBS settings for each patient are detailed in Supplementary Table S1.

**Table 1:** Demographical and clinical characteristics of all participants.

Characteristics	*N* = 29
Age(years)	
Mean ± SD	62.7 ± 7.4
Gender (male/female)	17/12
Education years	
Mean ± SD	10.9 ± 3.4
Disease duration (years)	
Mean ± SD	8.9 ± 3.2
Preoperative medication response (%)	
Mean ± SD	58.7 ± 14.3
MDS-UPDRS TD/PIGD ratio	
Mean ± SD	0.38 ± 0.33
LEDD (mg/day)	
Mean ± SD	666.5 ± 444.1
MMSE	
Mean ± SD	27.9 ± 1.8
HAMA	
Mean ± SD	13.6 ± 7.1
HAMD	
Mean ± SD	11.4 ± 6.2

MDS-UPDRS, Movement Disorder Society Unified Parkinson’s Disease Rating Scale; TD, Tremor-dominant; PIGD, Postural instability/gait disturbance MMSE, Mini-Mental State Examination; HAMA, Hamilton Anxiety Scale; HAMD, Hamilton Depression Scale; LEDD, levodopa equivalent dose; SD, standard deviation.

**Figure 3:**
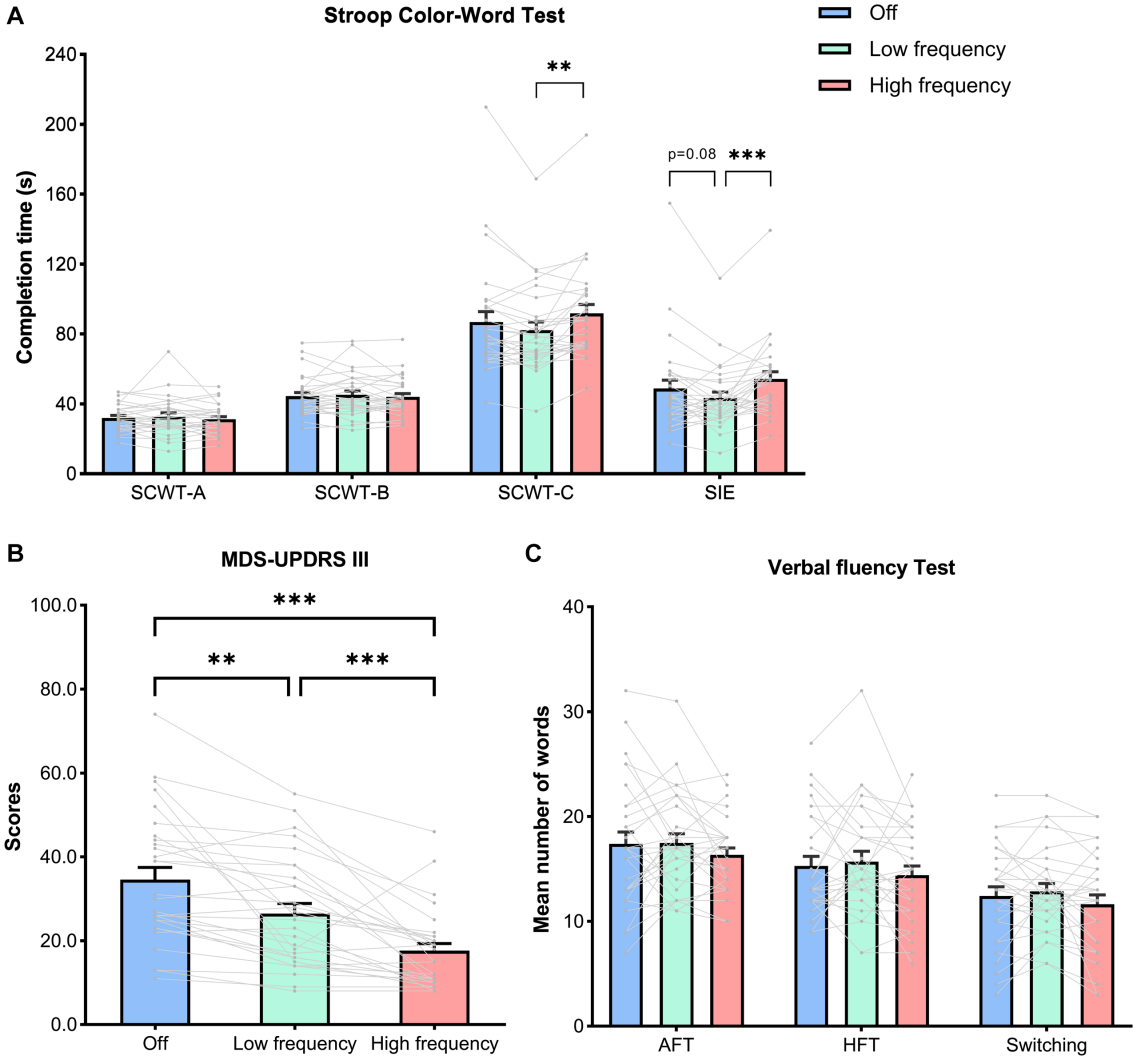
Neuropsychological and motor scores in stimulation states. Both individual and mean ± SEM of the all the patients are shown. **(A)** The completion time of the SCWT-A, SCWT-B, SCWT-C,as well as Stroop interference effects (SIE), calculated as follows: SIE = SCWT-C_time_ − [(SCWT-A_time_ + SCWT-B_time_)/2]. **(B)** The motor scores assessed by MDS-UPDRS III. **(C)** The scores of AFT, HFT and switching between two categories. **(A–C)** Analyzed by a repeated-measures analysis of variance with a Tukey correction. Abbreviation: SCWT, Stroop Color-Word Test; AFT, Animal fluency test; HFT, Household item fluency test; MDSUPDRS III, Movement Disorder Society Unified Parkinson’s Disease Rating Scale part III. *p* values shown represent an analysis of difference between two groups; *p < 0.05, **p < 0.01, ***p < 0.001.

Neuropsychological scores and motor scores at off (no stimulation), low frequency (5 Hz), and high frequency (130 Hz) are outlined in Table 2. For executive function, there was no difference among stimulation states in accuracy of SCWT-A, SCWT-B, and SCWT-C (*p* = 0.35, *p* = 0.37, *p* = 0.16, respectively). The completion time of the SCWT-A and SCWT-B were not affected by different stimulation states (SCWT-A_time_, *p* = 0.59; SCWT-B_time_, *p* = 0.62, Table 2). Of note, the comparison of stimulation states revealed significant difference in SCWT-C_time_ across stimulation states (*F* (2, 56) = 6.57, *p* = 0.005, η_p_^2^ = 0.19). Tukey *post hoc* analysis revealed that low frequency stimulation significantly decreased completion time of SCWT-C compared to high frequency (low frequency, 82.17 ± 24.80; high frequency, 91.72 ± 27.29, *p* < 0.001). Moreover, rmANOVA analysis indicated that there was significant difference in SIE among the mean of all the stimulation states (F (2, 56) = 7.99, *p* = 0.002, η_p_^2^ = 0.22). *Post hoc* analysis showed that the mean score of SIE in low frequency stimulation was significantly lower than in high frequency stimulation (low frequency, 43.29 ± 18.33; high frequency, 54.22 ± 21.85, *p* < 0.001). Although the difference did not reach statistical significance (*p* = 0.08), there was a trend towards decreased SIE in low frequency stimulation compared to no stimulation (low frequency, 43.29 ± 18.33; off: 48.67 ± 26.18, *p* = 0.08) (Figure 3A).

**Table 2:** Neuropsychological and motor performance in different stimulation states.

Neuropsychological test and motor function	Off		Low frequency		High frequency		rm ANOVA	
	Mean	SD	Mean	SD	Mean	SD	*F*-value	*p*-value
SCWT-A_accuracy_ (%)	99.86	0.52	99.93	0.37	99.72	0.88	0.77	0.44
SCWT-B_accuracy_ (%)	99.79	0.62	99.52	0.87	99.31	1.23	2.14	0.13
SCWT-C_accuracy_ (%)	97.79	2.58	96.69	2.58	96.62	2.78	2.07	0.14
SCWT-A_time_(seconds)	31.90	7.75	31.76	8.51	31.10	8.13	0.54	0.59
SCWT-B_time_(seconds)	44.34	11.34	45.03	12.21	43.90	10.68	0.46	0.62
SCWT-C_time_(seconds)	86.79	31.91	82.17	24.80	91.72	27.29	6.57	0.005^*^
SIE	48.67	26.18	42.39	18.33	54.22	21.85	7.99	0.002^*^
AFT	17.38	6.12	17.48	4.73	16.34	3.57	1.27	0.29
HFT	15.28	4.93	15.69	5.32	14.41	4.65	1.26	0.29
SFT	12.41	4.73	12.86	4.03	11.62	4.81	2.29	0.11
SDMT	28.34	11.24	29.48	12.41	28.97	9.92	0.34	0.68
DST-forward	5.67	0.89	5.64	1.04	5.52	0.71	0.46	0.62
DST-backward	2.86	1.16	2.98	0.94	2.88	1.01	0.31	0.72
JLO	18.41	4.57	19.72	4.84	18.76	4.68	3.00	0.07
MDS-UPDRS III	34.59	15.59	26.48	13.08	17.62	9.34	32.97	<0.001^***^

SCWT, Stroop Color-Word Test; SIE, Stroop interference effects, calculated as follows: SIE = SCWT-C_time_ − [(SCWT-A_time_ + SCWT-B_time_)/2]; AFT, Animal fluency test; HFT, Household item fluency test; SFT, Switching fluency test; SDMT, Symbol Digital Switch Test; DST, Digital Span Test; JLO, Benton Judgment of Line Orientation test; MDS-UPDRS, Movement Disorder Society Unified Parkinson’s Disease Rating Scale; SD, standard deviation; rm ANOVA, repeated-measures analysis of variance; Off, no stimulation; Low frequency, 5 Hz; High frequency, 130 Hz; *p < 0.05, **p < 0.01, ***p < 0.001.

However, no significant differences among stimulation states were found for other cognitive domains, including the language (AFT, *p* = 0.29; HFT, p = 0.29; Switching, *p* = 0.11, Figure 3C), attention (SDMT, *p* = 0.68), short-memory (DST-forward, *p* = 0.62; DST-backward, *p* = 0.72) and visuospatial ability (JLO, *p* = 0.07) (Table 2).

As expected, for motor score, results showed that high frequency stimulation (high frequency, 17.62 ± 9.34) significantly improved MDS-UPDRS III scores compared to either no stimulation (off, 34.59 ± 15.59, *p* < 0.001) or low frequency stimulation (low frequency, 26.48 ± 13.08, *p* < 0.001). In addition, low frequency stimulation also improved motor scores compared to no stimulation (*p* = 0.001) (Figure 3B).

## 4. Discussion

In this study, we conducted a prospective double-blinded randomized clinical trial that excluded the confounding effects of long-term high-frequency stimulation and anti-PD medications. Our study investigated the effects of low and high frequency bilateral subthalamic DBS on cognitive subdomains including executive function, language, attention, short-term memory, and visuospatial functions in PIGD patients during their initial programming. Here we found that bilateral subthalamic low frequency stimulation significantly improved executive function (SIE and SCWT-C) compared to high frequency stimulation. No difference in other cognitive subdomains was detected among low, high frequency and no stimulation. In addition, low frequency stimulation resulted in improved motor function compared to no stimulation and decreased motor function compared to high frequency stimulation.

Stroop Color-Word Test is extensively used to assess executive function, especially the ability to inhibit cognitive interference (Scarpina and Tagini, 2017). We found that low frequency STN-DBS resulted in improved executive function compared to high frequency STN-DBS, demonstrated by decreased Stroop effect (SIE and SCWT-C). Moreover, low frequency STN-DBS showed a trend of executive function improvement compared to no stimulation. STN is thought to play a central role in modulating responses during cognitive interference inhibition (Brittain et al., 2012), and elevated power levels in the STN LFOs were detected in these cognitive processes (Zavala et al., 2013, 2014; Zhang et al., 2022). Besides the STN, LFOs also coordinate the activities of distant cortical networks during cognitive function (Nácher et al., 2013), especially the medial prefrontal cortex (mPFC) (Kelley et al., 2018; Zavala et al., 2018). Inspiringly, subthalamic low frequency stimulation has been reported to improve cognitive control in PD patients (Kelley et al., 2018). Therefore, we uphold that subthalamic low frequency stimulation could possibly enhance executive function in PIGD patients via the mPFC-STN circuit.

In our study, we found that high frequency STN-DBS demonstrated a trend of executive function impairment compared to no stimulation. It is consistent with several studies (Scangos et al., 2018; Lam et al., 2021). Nevertheless, event-related high frequency STN-DBS has also been reported in increased low frequency power, disrupted conflict processing, and increased errors (Ghahremani et al., 2018). Several long-term follow-up studies showed high frequency STN-DBS could deteriorate executive function (Parsons et al., 2006; York et al., 2008). We think the discrepancy may due to the difference in stimulation duration. To sum up, we think subthalamic low frequency stimulation could possibly enhance executive function, and high frequency stimulation could possibly impair executive function, thus leading to the significant differences in SIE and SCWT-C between low frequency and high frequency stimulations.

We found no significant differences between low and high frequency STN stimulation in language, attention, memory, and visuospatial functions in PIGD patients during their initial programming. Nevertheless, previous studies showed low frequency STN stimulation moderately improved overall verbal fluency compared to high frequency stimulation (Wojtecki et al., 2006; Lam et al., 2021; Lee et al., 2021). We think the discrepancy may lie in patient selection. Patients recruited in the previous studies had relatively long-term (3 months to 2 years) high frequency stimulation (Wojtecki et al., 2006; Lam et al., 2021), and a decline in verbal fluency upon chronic high frequency STN-DBS has been reported (Parsons et al., 2006; Temel et al., 2006). In our study, patients were at their initial programming and their verbal fluency had not been impacted by the long-term high frequency stimulation.

As was expected, low frequency stimulation was worse than prevailing high frequency stimulation in motor function improvement. Nevertheless, to our surprise, low frequency stimulation resulted in improved motor function compared to no stimulation. On the one hand, this may due to the patients we included were PIGD subtype without obvious tremors, and previous studies mostly showed that low frequency STN-DBS lead to deterioration of tremor (Timmermann et al., 2004). On the other hand, the fact that the patients in our study were under the med-off condition, while earlier studies investigated the effects of low frequency STN-DBS under the med-on condition, may also account for the discrepancy.

Currently, there is a paucity of researches investigating the impact of DBS stimulation frequency on cognition in PD (Wojtecki et al., 2006; Scangos et al., 2018; Lam et al., 2021; Lee et al., 2021), and studies exhibit significant heterogeneity in terms of study design, neuropsychological testing, and outcomes. Therefore, larger sample size investigations are necessary to validate these findings and account for outcome heterogeneity. While low-frequency stimulation may not effectively manage motor symptoms in all PIGD patients, high-frequency stimulation remains the gold standard for DBS therapy targeting PIGD motor symptoms. However, our study and previous researches have demonstrated promising cognitive benefits associated with low-frequency stimulation. To optimize control over both motor and cognitive symptoms across various PD subtypes while minimizing adverse effects from single contact stimulation, future advancements in DBS technology should explore combining high and low frequencies through interleaving currents between multiple electrode contacts. Furthermore, when selecting the stimulation pattern, consideration should be given to pulse width and current intensity as they influence the effectiveness of DBS therapy. Accordingly, in line with the findings of this study, a combination of low-frequency and high-frequency stimuli can yield clinically beneficial effects on cognitive performance. Directional electrodes could potentially alternate between low and high frequency stimulations while providing additional programming patterns that better address the demands of improving both motor and cognitive function.

Several limitations must be acknowledged in this study. As previously stated, due to the limited sample size and the single-center nature, we believe that our results need to be carefully interpreted and further verified by studies with larger cohorts in the future. Second, although this study utilized a relatively longer stimulation time (22 h) compared to previous studies, the long-term effects of low frequency stimulation on cognition in a wider range of PD patients including PIGD subtype should be verified. Finally, it is worth noting that only the frequency of stimulation was altered while keeping the pulse width and current strength constant. Consequently, difference in total electrical energy delivered between low and high frequency may potentially affect changes in cognitive and motor function associated with PIGD.

## 5. Conclusion

In this study, we have discovered that low frequency stimulation during the initial programming of bilateral STN-DBS in patients with PIGD significantly enhances executive function compared to high frequency stimulation, while also improving motor function when compared to no stimulation. Our findings suggest a novel approach for DBS programming in PIGD patients, where low-frequency stimulation can be incorporated into their programmed mode. Further research is necessary to investigate the long-term effects of low-frequency stimulation. With the aid of variable frequency stimulation programming and advancements in directional electrode technology, we anticipate that both cognitive and motor symptoms in PIGD patients will be improved.

## Data availability statement

The raw data supporting the conclusions of this article will be made available by the authors, without undue reservation.

## Ethics statement

The studies involving humans were approved by Institutional Review Board (IRB) of Beijing Tiantan Hospital. The studies were conducted in accordance with the local legislation and institutional requirements. The participants provided their written informed consent to participate in this study.

## Author contributions

GQ and HX: conceptualization, methodology, data curation, formal analysis, investigation, validation, and writing—original draft. LS: conceptualization, methodology, investigation, validation, formal analysis, and resources. BZ: conceptualization, methodology, and supervision. YG and ZY: methodology, data curation, and formal analysis. YX, XZ, and YC: data curation and investigation. YJ: conceptualization, methodology, resources, supervision, and writing—review and editing. QZ: conceptualization, methodology, data curation, supervision, and writing—review and editing. JZ: conceptualization, methodology, funding acquisition, project administration, resources, supervision, and writing—review and editing. All authors contributed to the article and approved the submitted version.

## Funding

This work was supported by the National Natural Science Foundation of China (81830033 and 81671104).

## Conflict of interest

The authors declare that the research was conducted in the absence of any commercial or financial relationships that could be construed as a potential conflict of interest.

## Publisher’s note

All claims expressed in this article are solely those of the authors and do not necessarily represent those of their affiliated organizations, or those of the publisher, the editors and the reviewers. Any product that may be evaluated in this article, or claim that may be made by its manufacturer, is not guaranteed or endorsed by the publisher.
