# Supplementary material for: Unlocking potential: low frequency subthalamic nucleus stimulation enhances executive function in Parkinson’s disease patients with postural instability/gait disturbance
Source: Front Neurosci. 2023 Aug 30;17:1228711. doi: 10.3389/fnins.2023.1228711 (PMC10498764; doi:10.3389/fnins.2023.1228711)
Supplement: Supplementary file 1 [file Table_1.DOCX]

**Supplementary Table 1.** DBS stimulation settings of all patients.

| Patients | Activated contact | | Current intensity (mA) | | Pulse width (μs) |
| --- | --- | --- | --- | --- | --- |
|  | R | L | R | L | R/L |
| 1 | 3 | 6 | 1.0 | 1.0 | 60 |
| 2 | 2 | 6 | 1.5 | 1.5 | 60 |
| 3 | 4 | 8 | 1.5 | 1.5 | 60 |
| 4 | 3 | 7 | 1.0 | 1.5 | 60 |
| 5 | 2 | 6 | 1.5 | 1.5 | 60 |
| 6 | 4 | 8 | 1.7 | 1.9 | 60 |
| 7 | 4 | 8 | 1.0 | 1.1 | 60 |
| 8 | 4 | 8 | 2.5 | 2.5 | 60 |
| 9 | 2 | 6 | 1.5 | 1.5 | 60 |
| 10 | 2 | 6 | 1.4 | 1.5 | 60 |
| 11 | 3 | 7 | 1.5 | 1.5 | 60 |
| 12 | 2 | 6 | 1.3 | 1.3 | 60 |
| 13 | 4 | 8 | 0.6 | 0.7 | 60 |
| 14 | 3 | 7 | 1.5 | 1.55 | 60 |
| 15 | 2 | 6 | 1.4 | 1.6 | 60 |
| 16 | 3 | 6 | 1.7 | 1.8 | 60 |
| 17 | 2 | 7 | 2.1 | 1.45 | 60 |
| 18 | 4 | 7 | 1.5 | 1.5 | 60 |
| 19 | 3 | 8 | 1 | 1.8 | 60 |
| 20 | 3 | 7 | 2.25 | 2.1 | 60 |
| 21 | 2 | 6 | 2.15 | 1.65 | 60 |
| 22 | 3 | 7 | 2.4 | 2.4 | 60 |
| 23 | 2 | 6 | 2.7 | 2.5 | 60 |
| 24 | 2 | 8 | 2.25 | 1.95 | 60 |
| 25 | 3 | 8 | 2.25 | 2.25 | 60 |
| 26 | 3 | 7 | 2.5 | 2.6 | 60 |
| 27 | 2 | 7 | 2.2 | 2.9 | 60 |
| 28 | 3 | 6 | 1.95 | 2.15 | 60 |
| 29 | 4 | 6 | 2.15 | 1.9 | 60 |

R, right DBS lead; L, left DBS lead; Electrode contacts ranged from 1 (deep) to 4 (superficial) on the right side and from 5 (deep) to 8 (superficial) on the left side.
